# Asthma—Its Pathology, Treatment, &c.

**Published:** 1857-08

**Authors:** N. F. Powers

**Affiliations:** Thompson, Georgia


### ARTICLE II.

*Asthma—its Pathology, Treatment, &c.* By N. F. Powers, M. D., Thompson, Georgia.

To well understand this disease, a minute knowledge of the structures of the organs of respiration—a knowledge of their functions, of their muscles, nerves and connections, is indispensably necessary. Without such knowledge, a vague notion only can be had of the Pathology of Asthma.

The lungs perform a very important part in vitalizing and sustaining the system. See their structural arrangements—their adaptation to the offices they have to perform, as in excretion, in ærating the blood, and in causing it to flow in the minutest capillaries. All this, when viewed as a whole, must excite, in the mind of the philosopher, wonder and admiration. The lungs, with their appendages, form a beautiful and complicated mechanism. They never cease their movements after the first gush of air has filled them; they are never fatigued; they move day and night without our consciousness; continued are their movements until death whispers be still. Their respirations in health are as one to five pulsations of the heart, in Pneumonia as one to two, in typhoid disease one to eight. The time for a complete respiration is three and a half seconds. Dr. J. C. Draper states that by experiments he has ascertained the following interesting results:

1st. "On making sixteen respirations in a minute, and continuing the experiment for twenty minutes, the average of five different series of experiments gives 622 cubic inches of air expired each minute.

"2nd. On making six respirations in a minute, and continuing the trial for twenty minutes, the average of three series of experiments gives 511 cubic inches of air expired each minute.

"3rd. On making three respirations in a minute, and continuing the experiment for twenty minutes, the average amount of air is 1,079 cubic inches for the air expired in each minute." So that, if one respiration be made to five, the quantity of air consumed will be nearly doubled. The lungs then, in hurried respiration, do not consume as many inches of air, as when inspiration is deep and protracted.

The vesicles which are found in the parenchymatous structure, if spread out, would have a surface thirty-five times greater than the superficies of the whole body. Some eighteen thousand of these vesicles are clustered around each bronchi, so that there are in the lungs some six hundred millions.

Then what a great inlet are the lungs for the admission into the system of metaphitic exhalations, poisonous gasses, and all such agents as contaminate the blood, and produce a host of morbific influences, through the ganglionic system of nerves. Not only are the lungs the inlet—the point for the introduction of such agents as beget what is denominated the Asthma, but it is highly probable that the "Materies Morbi" of the greater number of the maladies of the human family find entrance here; even the ova of the parasites. It has been truly said, "Man's body is unquestionably a little world to many animals." Those found in the liver, in the bronchial glands, in the muscles, in the brain, and in the eye, and dermoid tissue, enter the system while floating in the atmosphere, and thus gather into the blood, while circulating, and become inhabitants of the body. Hence, causes may be compounded by being introduced simultaneously into the lungs, thus generating disease of a mixed character.

The term Asthma, as used by many, is indefinite. The word classically signifies difficult breathing. But Dyspnæa is a constant symptom of various diseases, such as Hydrothorax, Pleurisy, stertor in Apoplexy, Croup, and Emphysema. In all these, the harmony of the organic and functional activity of the respiratory apparatus is disturbed—the breathing is difficult; but nevertheless these diseases are not Asthma.

Asthma, in Dr. Cullen's system of nosology, is a genus of disease in class neurosis, and order spasmi. He points out three species, the spontaneous, the plethoric, and exanthematous. While others speak of the spasmodic, the pure nervous, the thymic, the humeral, the dry, the mucous catarrhal, latent catarrhal, acute, chronic, and congestive. Nearly every writer seems to have sought out some new name, and thus making confusion more confounded.

Many affections which have been denominated Asthma are only symptoms of disease, and should be regarded and treated as such by the practitioner. Dyspnæa may be a symptom of cardiac, gastric, cutaneous, intestinal and renal diseases. Nor is this surprising, when it is remembered that all the organs which are the seat of these diseases are so intimately connected with the lungs through the ganglinnic system of nerves· From these causes, we find those persons who are predisposed to Asthma, often labor under dyspepsia, diarrhœa, cardiac affections, gastric, intestinal, renal and cerebro-spinal, hepatic and cutaneous diseases. This is a consequence of the hypersensitiveness of the eighth pair or par vagum nerve.

Under the influence of opium, a mere touch upon the skin will often produce spasm, and again, cold water thrown on the face will relieve syncope. These results take place from the impressions transmitted through the skin nerves to the nerve centres.

Idiopathic Asthma is used here to signify a difficulty of breathing, originating in some of the tissues of the organ itself, or in the non-fulfilment of some of the respiratory functions. It is not dependent on some other organic disease; or in other words it is not symptomatic. This is the variety of Asthma now under consideration. It may be paroxysmal, intermitting and periodic. An attack predisposes to its recurrence under similar circumstances or exciting influences.

Asthma is essentially a disease of the bronchial tubelets and vesicles affecting various other organs in their functional performances. This is a natural sequence, as healthy respiration is so necessary to the support and continuance of the vital phenomena; such as the circulation of the blood, the assimilation of the Chyle, to the calorification of the whole system, and the renewal of the tissues. For this purpose, atmosphere must be admitted into the lungs, through the bronchi, else the blood cannot be ærated. The atmosphere is caused to pass into the lungs by the action of the thorax and diaphragm, through the bronchial tubelets, into the pulmonary vesicles. As has already been stated, these vesicles are very numerous, and are divided into groups denominated lobules, which are independent of each other. The air cells or pulmonary vesicles are also independent of each other, being connected only by their pedicles. The bronchial tubelets, which admit the atmosphere, as has been clearly demonstrated, are made up of mucous-membrane, cellular tissue, longitudinal and circular fibres. These fibres are contractile, or in other words they are muscular and may be spasmodically affected.

Now, Asthma *originates primarily in the mucous membrane of the air breathing passages, generating an impression, which through the nerves is reflected on the muscular fibres, causing their contraction or spasmodic action.* The spasm of the longitudinal muscular fibres of the bronchial tubelets, lessens their calliber, diminishes the quantity of supplied atmosphere, thus congests the venous capillaries of the pulmonary tissue, lessening their capacity, so that the blood is not normally ærated. These effects and sequences, when taken together, is Asthma, which takes place through the agency of the nerves of respiration These are derived principally from the Pneumogastric and sympathetic nerves. None of the fibres of the latter are per se motor or sensory, or do its ganglia produce reflex action. The agency which it exerts is due to its association with the cerebro-spinal nerve fibres. It lessens or increases by the influence it exerts over the contractility of the heart, arteries, and capillary nutrition and secretion. And in this way may, through sympathy, generate pulmonary diseases. The pneumogastric is the principal nerve of respiration, though the sensory nerve of the face is derived from the 5th pair, which is made manifest by the action of cold air or water. Nor is it restricted to cold air or water, as is proven from the beneficial effects of titillation and other kinds of irritation of the skin, in keeping up the respiratory movement in Narcotism. The Pneumogastric, in fulfilling the respiratory functions, associates with the sympathetic and spinal accessory, and thus becomes bi-functional, and through it the want of air is felt in Asthma.

Even when the air is admitted into the lungs, if its oxygen is not taken up by capillaries, the supply of oxygenated blood must be deficient in carrying on the various processes of vitality. The pulmonary capillaries spread out their fimbriated extremities into an expanded net work, which surrounds each air cell, and ramifies into the parenchymatous structure of the lungs. Some think that in this structure carbonic acid gas is formed, others that it is a secretion of the mucous membrane. But neither of their positions are true, as it is generated in the blood itself, at least in all vertebrated animals. The capillaries are separated from the air cells by a thin film or membrane of great tenuity, so that the exchange of the different gases takes place rapidly through it. This exchange is entirely of a physical character, in which the nervous systems are not engaged. In Asthma, the exchange of gases is deficient, in consequence of the want of a normal supply of atmosphere; the contraction of the air tubes preventing its admission. Consequently, there must be a partial stagnation of blood in the pulmonary capillaries—a lessening of the propelling power of the heart; hence it flutters, throbs and palpitates—a diminishing of the primary cause of the circulation and the vital stimuli, and an arresting partially of the excretory action of the lungs, which has a morbific influence on the liver and kidneys. As the lungs and liver are developed in an inverse proportion, the inference is, that when respiration is interrupted for a length of time, the liver must be affected to a greater or less degree. If respiration is lessened, the functional activity of the liver must be increased, as well as that of the kidneys and skin. Consequently, diseased livers and kidneys are often found in the Asthmatic, and as an effect of these disorders may follow a host of other diseases. To comprehend these various results, the sympathies, both contiguous and continuous which may be aroused by impaired respiration is highly interesting and important to the physician. It seems that without it he must be a mere groper in the dark —an empiric. "To be a good doctor, one must be a great physiologist."

When spasms or contractions take place in the bronchial tubelets, as in Spasmodic Asthma, they are not, in consequence of disease of the excito-motor or excito-secretory system of nerves, for then the disease would be more permanent and only a symptom. According to Sir Marshall Hall's theory, spasms as well as convulsions are not local, but originate in the diseased state of the spinal or excito-motory system. But with due reference to the high authority, perhaps it may be asserted that in the majority of the instances in which spasms take place, there is previously no diseased action existing in the spinal system of nerves. If spasms supervene in dentition—in the bronchial muscular fibres, as in Asthma—in spasms of the stomach or intestines, the prima causa acts directly on the fimbriated extremities of the nerves involved. The cause in lentition is the teeth acting on or irritating the branch of the fifth pair of nerves, while that of the stomach, intestines and lungs is the pneumogastric and spinal nerves. Hence the prima causa, an irritant—an excitor of the motory system,—begets spasms through the reflex functions of the nerves, so that in Asthma the sensitive branches of the fifth pair, become first excitors of the spinal nerves, which are involved in sneezing, producing action in the muscles of respiration, and a flow of mucus, from the anterior and posterior nares. The same principles are involved in reference to the cause and reflex action throughout the list of diseases, which are denominated spasmodic. The motor-excitor and motor-secretory generate influences in accordance with their functions, not that they are the primary seat of disease, but because they are excited into action from the diseased condition of the tissues to which they are distributed. This tissue in Asthma, and intestinal diseases, is the mucous membrane. Dr. Campbell of Augusta, says: "Local irritations can, through this system, (excitor-motor) produce convulsions by the reflex functions of the nerves, the sensitive branch of the fifth pair becoming excitor to the motory spinal nerves, and so we may justly infer do these same branches of the fifth pair, under certain circumstances, become excitor to the secretory filaments of the sympathetic." In the same manner may the secretory filaments be affected in Asthma. Consequently all the muscles of respiration as well as those organs of secretion and excretion are involved in Asthma.

There is but one variety of Asthma, which is not a symptom of any other disease. What is called Asthma by many is only a Dyspnœa. Asthma, it is true, may occur simultaneously with other ailments, but these diseases do not bear the relations of cause and effect to each other.

The symptoms of Asthma are so characteristic, that it may be readily distinguished from other diseases. The premonitory, when superinduced by the inhalation, through the nostrils of irritants, are ticklings of the anterior nares, an irresistible desire to sneeze, attended with a rapid secretion of mucus, from the pituitary membrane—hypersensitiveness of this membrane, gradually extending through the posterior nares over the fauces and larynx—profluvia of mucus not very tenacious, resembling that in common catarrh—irritation of the bronchus—labored and distressing cough—eyes red and suffused; the mucous, especially that from the bronchial tubes becomes more and more tenacious, producing a titillating sensation in the throat so as often to superinduce emesis. It is transparent when held on the tip of the finger, tremulous, looking like a gelatinous mass. It is expectorated with difficulty, and is forced up from the bronchial tubes, which always gives partial relief. All the above named symptoms may be brought on by the mere inhalation of a small particle (so minute that it cannot be seen) of "Ipecac." After the vesicles themselves become involved, then symptoms become prominent. The patient complains of stricture across the chest, extending around the entire thorax, or he feels as if he is tightly bound with strong cords, so that he feels that he only has the power of elevating the shoulders and throwing back the elbows, without the ability of extending the ribs, so essential to a complete inspiration.

This spasm of inter-costal muscles extends even to those of the floating ribs. The spasm of the muscles supervening, the sibilent rale is heard in the bronchial tubelets. From physical signs it is now presumable that the longitudinal fibres of each bronchi and vesicle are spasmodically affected, so that the escape of air at each expiration produces a wheezing rattling sound, resembling the air passing through tubes whose ends are partly covered by a tenacious mucous; which rises and falls corresponding to the inspirations and expirations. When the muscular fibres of the bronchi and the respiratory muscles of the chest becomes affected, then comes the horror of the disease; only air sufficient, though barely, for life is admitted—none to spare—none to waste, but still a constant and invincible desire to gasp it, cool and fresh, to take deep and long inspirations, but then the disease, like the close hug of death holds—binds in cords too strong to be broken. The eyes are now red, suffused, and protruded, as one about to die from asphyxia, the blood vessels on the forehead and face are turgid, looking full and prominent. The complexion is often dark, the lips swollen and purple. The whole face sometimes in color, looks like one suffering from urticaria, the feet and hands are colder than natural. The pulse in the robust is frequent and full—in the old small and compressible—blood when extracted is darker than in health. The temperature is lowered from 18°to 20°; the kidneys secrete rapidly a limpid urine, begetting an urgent desire to micturate, when cystic contractions are rather spasmodic. This is even felt in the larger intestines, so that the desire to visit the temple of cloecina often comes on unexpectedly, and is unusually hasty in its demands. Speech is difficult, not from a loss of power to articulate, but from the want of time. The easiest, if there can be any, is the semi recumbent position, with the head and shoulders supported by pillows—the former reclining so as to elevate the chin, the elbow thrown back and the sternum raised, so as to give, by position, as much freedom as possible to the organs of respiration. The duration of the paroxysm is uncertain.

The time of attack is variable according to the causes, peculiarities of patient, &c. In the majority of instances, it comes on about 2 o'clock at night. If produced by the inhalation of Ipecac, the fumes of a sulphur match, the "hay odor," or the passing of a cloud surcharged with electricity, then the attack follows immediately after the action of the exciting cause. But even when this is the case, if the disease after the first paroxysm returns, it will come in the night, the patient suffering all the distress of Asthma, when waking from his first sleep. Even such attacks as the above, although produced by odors, &c., and periodical, continuing as a quotidian for four or five days, and in some rare cases even for ten weeks. Though in the protracted cases, the patient is never entirely free from uneasy sensations and whizzing sounds in the lungs.

Some writers have asserted that Asthma is a disease of adult age, but this is not the experience of all. It may occur in infancy. One patient seven years old, who came under the observation of the writer, suffered from a severe attack. Not only did she have Asthma, the respiratory functions being primarily affected, but there was also spasms in the muscles of the face and eyes, so much so that permanent strabismus was superinduced, and strange to say hemiopia is now one of its sequences.

When Asthma has been severe and of long standing, the patient is much emaciated, indicating a deficiency in the nutritive process, the shoulders are elevated and fall too far forwards, the countenance has a peculiar expression, marked by the lines of anxiety, of restlessness. If the disease is of "long standing," the heart, the liver, the kidneys and skin become morbidly affected.

The diagnosis in this disease is not difficult. There are many diseases which have dyspnea as a symptom, but one who has ever seen or studied the pathology of Asthma, cannot make such a blunder as to confound the symptoms of other diseases with those of Asthma.

To diagnose properly is a matter of the highest importance, but especially in Asthma.

The wise physician makes out his diagnosis from signs exhibited by the countenance; in the peculiarities of attitude; from the condition of the digestive organs; from the various attitudes of the body; from the manifestations of the nervous system; from the normal and abnormal condition of the circulatory apparatus; the respiratory apparatus; the emunctories of the body; from the lymphatic system, and from the organs of secretion: as for instance, if there is an affection of the respiratory apparatus, manifested by panting—short, labored and anxious respiration. If there is wheezing, if there are physical signs indicating contraction of the bronchial tubes, why the diagnosis must be pronounced Asthma.

A few of the diseases, which in their development resemble some of the features of Asthma will now be pointed out.

In hysteria, the glottis is often spasmodically affected, resulting in dyspnœa, which to a careless observer might be diagnosed asthma, but then the lazy movements of the hysteric, the horizontal posture of the patient, when compared with the hasty movements, and semi recumbent posture of the Asthmatic, as well as the hasty efforts after air—the almost grasping with the hands for it, or the leaning forward with elbows on the knees, the extreme distress, the urine clear, abundant, especially after retiring to bed, limpid, the sputa clear, tenacious, but few air vesicles to be seen in it all give characteristic features, well known to old acquaintances.

Besides in all laryngeal affections, the patient points out the throat as the seat of the disease, by handling it, as if to relieve or enlarge the respiratory inlets. In such affections even the child will grasp its throat with its little hands. Besides these, there is another diagnostic sign, not common in laryngeal affections, but peculiar to Asthma, a wheezing puring sound can be heard by auscultation.

The sounds in Bronchitis are somewhat after the same note, but they can by an erudite ear,be readily distinguished,although to the novitiate, they may be considered identical. Indeed one may be attacked by Asthma while suffering from Bronchitis, which will render the signs similar and difficult to be diagnosed. In bronchitis there is fever; the attack takes place gradually, while in asthma it comes on suddenly and there is no fever. The chest in bronchitis does not feel as if bound by chords—the chest can be expanded to the utmost. Dyspnea is a common attendant on many other diseases of the thoracic and abdominal viscera, which, when the lungs become involved, and then are taken, in an isolated form may resemble Asthma, but all these diseases can *now be readily* diagnosed, and their etiology well understood by the assistance of auscultation and percussion. Besides the above mentioned signs, there are many others, such as those of the countenance and of the thorax. This can be seen in the contracted chest and the elevated shoulders. Besides it is surprising that Asthma is not more common, when the mal-formed chest of mothers of the present day is scanned. Perhaps this is the prima causa of Asthma being so common in the present day. It might be called the mother's folly, resting as a curse upon the child. The females of the past age thought they would improve the figure the wise God gave them. They preferred the wasp or hour glass shape, to those models of perfection, the venus of milo and venus de medici. Powers, our illustrious countryman, says: "Eve is an old-fashioned lady, and not so well formed or attractive as her grand-daughters, at least so think some of them. She wears her hair in a natural and most primitive manner. * * * * Her *waist is quite* too *large for our modern notions of beauty.* But Eve is very stiff and unyielding in her disposition. She *will not allow her waist to be reduced* by *bandaging, because she is far more comfortable as she* is, and besides, *she* has some regard for her health, which might suffer from such restraints on her lungs," &c.

*Causes of Asthma.*—These are varied and numerous, and act on mucous membranes, on the skin, and through the nerve centres. Without a knowledge of the causes of Asthma, its treatment must be mere empyricism. For classification, these may be divided into the predisposing or remote, the proximate or pathological. The latter have been already pointed out. The former give origin to those changes so essential to the action of the proximate causes of Asthma.

The exciting causes of Asthma are such as develope the disease after the remote has established a basis for action. Although the predisposing cause of Asthma may exist, nevertheless an attack may never supervene, if the pathological do or has not existed. The exciting cause without the predisposing may be simple and harmless: as for instance the sudden vicissitudes of temperature—a current of cold air coming in contact with the neck and chest, the inhalation of offensive substances, deprivation of sleep, anxiety of mind, the excitement of the moral feelings, the indulgence in wine drinking, and darkness. All these may be exciting causes, provided the predisposing have prepared the system for their reception.

The remote causes of Asthma may be divided into the internal and external. The internal are such as have been already mentioned and also include malformations of the chest, and of inlets of the thorax, hereditary transmissions, hypersensitiveness of the pulmonary mucus membrane, which renders it very susceptible to morbid influences. The external consist of such as odors, gasses, cold air, a warm damp atmosphere. Nor is it singular that the latter should produce asthma, when it is remembered that the skin is one of the greatest emunctories of the system, and eliminates eleven while the lungs eliminate only seven grains, or in other words according to Sequin's experiments, the loss through the skin is, during the day, two pounds and three quarters. Now a moist warm atmosphere must check the exhalation of the skin, the elimination of effete matter, which may cause Asthma in the susceptible or predisposed. Hence the majority of Asthmatics often complain of oppression when such is the state of the atmosphere.

Another external cause is Ozone. Which is a peculiar modification of oxygen, through the action of electricity. Perhaps it may be called the oxide of electricity. This change in oxygen, or the existence of ozone is quickly perceived by the Asthmatic. He is so sensitive that its presence awakens him from deep sleep when an electric cloud passes—he feels restless when the thunder sounds and the lightning flashes—not from fear or dread, for often when he has not seen, being asleep, he finds himself, on waking, setting up in bed, wheezing and gasping after air. He becomes a sort of living barometer. Consequently when influenzas prevail, Asthmas are common. Besides, Asthmas, perhaps from the same causes, are common in localities peculiarly adapted to the prevalence of remittent and intermittent fevers, or periodical disease of malarious origin. Asthma is a stranger like intermittent fever in the midst of populous cities, where is nursed to perfection the germs of typhus. The existence of these causes may be due to the presence or absence of ozone. If in excess it hastens; if deficient it prolongs putrefaction. So that if ozone is generated in large quantities, it rapidly destroys putrifying organic matter, so as to render it partly innocuous, or if not generated it prolongs the time of the putrifying process, so as to scatter abroad pestilence and death.

The predisposing causes may be common or general. The general may be such as act on a number of people at the same time or upon all the inhabitants of the same community. Ozone may be a general cause, developing Asthma in some and influenza in others. The odor of hay, the falling of autumnal plants, &c., may on the predisposed become a general cause.

If Asthma has a specific cause it is not yet known. There are cases which have been developed by the inhalation of powdered Ipecac, without the patient having a hereditary predisposition. It is well known that Ipecac is an irritant to all the mucous membrane. This cause of Asthma may be come predisposing, and in the same way ozone may be predisposing and exciting.

*Treatment.*—To wisely adopt in every instance a remedy to disease in all its protean forms, is of difficult attainment to the physician. But the perfection of medical skill must be in truthful diagnosis, and in the finding out an individual remedy for every individual disease. To do this it is necessary to isolate every disease, to understand the functions of every organ with their physical relations, the manner in which their functions are performed, whether they may be morbid or healthy, to have an absolute idea of disease, and be enabled to trace it to its remotest, as well as minutest bearing. When this is done the remedy may be readily selected, more easily found, and when discovered better understood. A disease to the physician should indicate the remedy, if it have any.

To the profession, as yet, the treatment of Asthma is vague and uncertain. This may be in consequence of its pathology not being understood, and its cause not known. To treat symptoms in accordance with *their* indications would comprise nearly all that is known of its medication. That is, if the pulse is full and bounding, the face flushed, &c., and the patient young and robust, why the lancet must be used. So with all other symptoms, let them be relieved by their appropriate and customary treatment.

For convenience and proper classification, the treatment of Asthma may be divided, first into that which is done during a paroxysm, and secondly what is done in the interval for its relief.

As Asthma is a spasmodic disease, perhaps it will be well to notice what anti-spasmodic will be the most useful in the treatment of the disease during a paroxysm. These remedies are generally taken into the stomach. Their influences are then transmitted to other parts through the agency of the nerves, and as these act as curative so may others act as morbific agents, and in the same way the mucous membrane of the lungs may be a nucleus for the germs of Asthma. These germs may be different—hence we may have different *types* requiring different remedies. We may see this truth illustrated in the variety of idiopathic fevers. Each kind or variety depends on a specific, sufficient predisposing cause. These differences depend on a variety of circumstances, such as elementary combinations, states of the atmosphere, moisture, heat, light, and electricity. Predisposition, when these agents act, frequently gives the features and individuality to disease. Since the types of Asthma may be different, remedies should be directed to the diseased organs, and to their real conditions; ever remembering in our treatment the spontaneous changes which must occur, while the disease progresses or declines, and that these are modified by remedial agents, and by sympathies with other organs.

The anti-spasmodics used in Asthma, embrace nearly all of the narcotics. But only a few of these will now be pointed out, as it is not thought that any of them act *as curative agents*, but only as palliatives. Opium and its various preparations are always useful. These should be administered after the action of an emetic, if the stomach is full. The anodyne preparations may be combined with Tinctura valerian, or Nitrous Ether. These remedies will lessen the irritability of the mucous membrane of the pulmonary tubelets, and will relax the pulmonary constriction.

For certain forms of Asthma preparations of the Atropa Belladonna, and Datura Stramonium have been highly extolled, especially the latter when smoked like tobacco. They both dilate the pupils, and the former produces excessive dryness of the throat, which is very disagreeable. Some had rather have Asthma than to suffer the effects of these poisons. Stramonium lessens the contractile power of the muscular fibres of the bronchi, as proven by the experiment of Williams on the lungs of animals. These muscular fibres when so relaxed are not susceptible to the action of galvanism. Again others are relieved by the use of tobacco.—Smoking the pipe is the most common and preferable method, but this soon loses its medicinal virtues. The tincture of Lobelia Inflata is spoken of highly by many as an anti-spasmodic in Asthma, but if it has any virtues they are due to its effects as an emetic. The Tart. Antimony and Ipecac is preferable.

There are many other remedies which may be used during a paroxysm, by far superior to the foregoing.

In the first place, immerse the feet and legs up to the knees in hot water, make cold applications to the head, and mustard to the spine, and if an emetic is demanded administer a warm solution of chloride of sodium, or common salt. If the patient is not relieved by this treatment, recourse must be had to other palliatives, such as the inhalation of chloroform. Sulphuric Ether and the deutoxide of Nitrogen. If these fail, or if they cannot be had, make a solution of the Nitrate of Potassa, in which dip the white tissue paper, dry it, tear thin slips, so that they may be burnt without making much smoke, set this on fire and draw it through or before the opened mouth while burning, and take deep inspirations, so as to inhale the smoke. Often this will relieve sooner than anything else, especially when expectoration is deficient.

The inhalation of vapor of water, medicated with Tannin, Creosote, and Chloroform is often very soothing, diminishes the erethism of the nervous system, and renders expectoration less difficult. The treatment, during the interval has to be varied according to circumstances. If the patient be old, feeble, and ossification of the cartilaginous portion of the ribs has taken place, but little can be done. Such medicines as may tend to strengthen should be used ; vegetable tonics, such as Tincture Cinchona, Gentianæ, &c., combined with gentle expectorants. But in many instances the mineral tonics are preferable, especially if the patient is very pale.

If the paroxysms supervene, as they generally do, periodically, S. quinine and Fowlers solution of Arsenic should be administered, as in intermittent fever. No remedies according to the experience of the writer, have such controlling influence over the disease, which prove to his mind that the causes of Asthma, not *symptomatic Asthma*, are nearly identical with those ot the periodical fevers. These remedies may be combined with tonics, expectorants, and anodynes as symptoms require.

The inhalation of medicated vapor generated from liquids or solids, are often found beneficial. These vapors may be made in various ways. One of the most convenient is to place the substances from which the vapor is to be generated in the "inhalator," and then heat "pro re nata." The vapor of Iodine, Stramonium and Aconite, well mixed with the atmosphere, will, it is believed, by protracted use, remove the hypersensitiveness of the mucus membranes of the bronchi, or the erethism of the respiratory nerves. The combination of creosote and many of the narcotic liquid extracts may be combined with other medicinal agents in gaseous form to suit the condition of each patient, according to their common therapeutic effects, and the wisdom of the practitioner.

The writer once knew a physician to administer a teaspoonful of the Spirits Turpentine mixed with a mucilage of gum acacia, during a paroxysm, which gave great relief in some ten minutes. The patient being a doctor asked his physician why he gave the Turpentine; his answer was, "I gave it to an old woman in a similar situation, when I had nothing else to give, and it relieved her. I now give it to you because it was a benefit to her." I have since found it an excellent remedy, during the paroxysm, for the old, besides it may be given occasionally with good results in the interval. Its therapeutic action is perhaps similar to creosote, and besides it has a direct diuretic tendency and acts on the superficial capillaries and also on the dermoid tissue.

The Asthmatic should use, during the interval, the cold bath, or sponge the chest and body every morning with cold water, and rub the surface of the entire body until the skin is reddened. If the cold should produce, (from debility,) too great a shock, use the flesh brush, and only place, on rising out of bed, the feet in a basin of cold water. This alone will lessen the susceptibility to cold. A small blister on the cervical vertebræ, kept vesicated by repeated applications, and the application of croton oil to the breast and lower portion of the throat, are by some considered highly curative, but this notion does not correspond with the experience of others.

Electricity has been highly recommended by some of the most learned of the profession; but others again who are Asthmatics, experience very disagreeable sensations when even in the vicinity of a large electric machine when in operation—though electricity in some of its forms may be curative, as for instance, galvanism, though its action must be slow and only alterative in its character. The manner of applying it may be seen, by referring to Dunglison on new remedies.

The writer does not believe that in one sense Asthma is curative, in another he believes that it is. Asthma is cured in the same sense that intermittent fever is; though the predisposition and tendency to recurrence is greater in the former than in the latter.

The writer, in conclusion, will say that what has been suggested as curative agents have been seen and felt. Should any have discovered a permanent cure—a remedy better than any suggested, why suffer me to beg you to let it be *immediately known.*

